# Reversal of neurological deficits by painless nerve growth factor in a mouse model of Rett syndrome

**DOI:** 10.1093/brain/awad282

**Published:** 2023-08-26

**Authors:** Alexia Tiberi, Giulia Borgonovo, Giovanna Testa, Paola Pacifico, Ajesh Jacob, Mariachiara Di Caprio, Valentino Totaro, Mariantonietta Calvello, Antonino Cattaneo, Simona Capsoni

**Affiliations:** Institute of Neuroscience, CNR, 56124 Pisa, Italy; Bio@SNS Laboratory of Biology, Scuola Normale Superiore, 56126 Pisa, Italy; Bio@SNS Laboratory of Biology, Scuola Normale Superiore, 56126 Pisa, Italy; Bio@SNS Laboratory of Biology, Scuola Normale Superiore, 56126 Pisa, Italy; Bio@SNS Laboratory of Biology, Scuola Normale Superiore, 56126 Pisa, Italy; Bio@SNS Laboratory of Biology, Scuola Normale Superiore, 56126 Pisa, Italy; Bio@SNS Laboratory of Biology, Scuola Normale Superiore, 56126 Pisa, Italy; Bio@SNS Laboratory of Biology, Scuola Normale Superiore, 56126 Pisa, Italy; Bio@SNS Laboratory of Biology, Scuola Normale Superiore, 56126 Pisa, Italy; Bio@SNS Laboratory of Biology, Scuola Normale Superiore, 56126 Pisa, Italy; Rita Levi-Montalcini European Brain Research Institute (EBRI), 00161 Roma, Italy; Bio@SNS Laboratory of Biology, Scuola Normale Superiore, 56126 Pisa, Italy; Section of Human Physiology, Department of Neuroscience and Rehabilitation, University of Ferrara, 44121 Ferrara, Italy

**Keywords:** neurodevelopmental disorders, neurotrophic factors, neurotrophin-based therapy, immunomodulation

## Abstract

Rett syndrome is a rare genetic neurodevelopmental disease, affecting 1 in over 10 000 females born worldwide, caused by *de novo* mutations in the X-chromosome-located methyl-CpG-binding protein 2 (*MeCP2*) gene. Despite the great effort put forth by the scientific community, a therapy for this devastating disease is still needed. Here, we tested the therapeutic effects of a painless mutein of the nerve growth factor (NGF), called human NGF painless (hNGFp), via a non-invasive intranasal delivery in female *MeCP2*^+/−^ mice. Of note, previous work had demonstrated a broad biodistribution of hNGFp in the mouse brain by the nasal delivery route.

We report that (i) the long-term lifelong treatment of *MeCP2*^+/−^ mice with hNGFp, starting at 2 months of age, increased the chance of survival while also greatly improving behavioural parameters. Furthermore, when we assessed the phenotypic changes brought forth by (ii) a short-term 1-month-long hNGFp-treatment, starting at 3 months of age (right after the initial presentation of symptoms), we observed the rescue of a well known neuronal target population of NGF, cholinergic neurons in the medial septum. Moreover, we reveal a deficit in microglial morphology in *MeCP2*^+/−^ mice, completely reversed in treated animals. This effect on microglia is in line with reports showing microglia to be a TrkA-dependent non-neuronal target cell population of NGF in the brain.

To understand the immunomodulatory activity of hNGFp, we analysed the cytokine profile after hNGFp treatment in *MeCP2*^+/−^ mice, to discover that the treatment recovered the altered expression of key neuroimmune-communication molecules, such as fractalkine.

The overall conclusion is that hNGFp delivered intranasally can ameliorate symptoms in the *MeCP2*^+/−^ model of Rett syndrome, by exerting strong neuroprotection with a dual mechanism of action: directly on target neurons and indirectly via microglia.

## Teaser

Tiberi *et al*. show that intranasal delivery of a ‘painless’ variant of human nerve growth factor (hNGFp) reduces behavioural impairments in a mouse model of Rett syndrome via effects on cholinergic neurons and microglia. hNGFp rescues the expression of key cytokines, potentially restoring neuroimmune communication.

## Introduction

Rett syndrome (OMIM identifier #312750) is a severe neurological disorder affecting development and function in females, at a rate of 1 in every 10 000 live births.^1,2^ In the majority of cases (90–95%), patients diagnosed with classic Rett carry loss-of-function mutations in the X-linked methyl-CpG binding protein 2 (*MeCP2*) gene.^1,2^ Because of its chromosomal position, random X chromosome inactivation renders female patients somatic mosaics, expressing with varying degrees mutant and normal MeCP2. Rett syndrome is not immediately evident postnatally: patients experience 6 to 18 months of seemingly normal development. After that, Rett-causing mutations lead to a critical developmental regression, during which affected children lose acquired speech and hand skills and they begin manifesting stereotypic movements, gait abnormalities, seizures and mental retardation.^3^

The neurotrophin nerve growth factor (NGF)^4^ has been previously linked to Rett syndrome because of its reduced levels observed in the brain and blood of patients.^5^ NGF is a potent neurotrophic factor for a number of peripheral and central neuronal cells.^6,7^ Indeed, NGF is required for the survival of the broad projecting population of basal forebrain cholinergic neurons, the very classical and well validated target of NGF, bearing the marker choline acetyltransferase (ChAT).^8,9^ Of note, ChAT^+^ neurons are decreased throughout the forebrain of patients affected by Rett syndrome.^10^ Notably, NGF also acts on a number of non-neuronal cells. Among these, recent work has revealed that NGF acts on brain microglia cells, steering them toward a neuroprotective phenotype.^11^ It is noteworthy that, though a more causative role for microglia in Rett pathogenesis has been revisited time and again due to some controversy,^12,13^ recent work has reiterated how this cell type could contribute to the disease.^14-16^ Thus, altogether these data provide a solid rationale for exploring an NGF-based therapy for this neurodevelopmental disease.

Despite its general therapeutic potential, the clinical applications of NGF in neurological diseases^17^ have been hampered by the fact that NGF induces severe pain, through its physiological pain-sensitizing actions.^18,19^ To overcome these hurdles, we have developed human NGF painless (hNGFp), a ‘painless’ variant of NGF with a >10-fold reduced pain-sensitizing activity tested in a number of different pain assays.^20,21^ From a pharmacological point of view, hNGFp is a tropomyosin receptor kinase A (TrkA)-biased agonist, in that it binds TrkA with identical affinity to that of wild-type NGF (wtNGF), while it binds p75^NTR^ with a 200-fold reduced affinity, thus shutting down pain-related signaling.^22^ The neurotrophic and neuroprotective properties of hNGFp are identical, if not superior in some cases (due to the lack of p75^NTR^ signalling), to those of wtNGF.^22^ We have demonstrated that hNGFp can be effectively delivered to the brain using an intranasal route of administration,^20,22^ with wide anti-neurodegenerative effects in three mouse models of Alzheimer’s disease.^20,23^ hNGFp owes its robust and consistent effects to its dual mode of action: it can (i) positively influence cholinergic neurons of the basal forebrain; and (ii) exert a broad neuroprotective activity on diverse neuronal populations in the CNS via microglia. Indeed, we and others have demonstrated that wtNGF and hNGFp have beneficial immunomodulatory properties that can affect microglial cells.^11,23-26^ Thus, we postulated that hNGFp might represent a potential therapeutic agent for Rett syndrome.

In this work, we aimed at testing the hypothesis that hNGFp might be a valuable therapeutic candidate for Rett syndrome, employing intranasal administration to female *MeCP2*^+/−^ mice. This mouse model fully recapitulates symptoms of Rett syndrome and is widely used in the literature to study the mechanisms underlying this disease.^27^ In particular, female *MeCP2*^+/−^ mice begin to show a diseased phenotype around 3 months of age, peaking after 5–6 months. We first performed (i) a lifelong intranasal treatment with hNGFp, starting at 2 months of age (before the appearance of behavioural deficits) up to a humane end point, in order to evaluate the ability of the neurotrophin to prevent the manifestation of symptoms and to appraise survival. This treatment was able to increase lifespan of *MeCP2*^+/−^ mice, while concomitantly improving Rett-relevant behaviour. In a second cohort of animals, we assessed the cellular and molecular effects of (ii) a short-term 1-month-long intranasal treatment with the neurotrophin, starting at 3 months of age, after the presentation of the first symptoms. Here, we observed a rescue of ChAT expression in the cholinergic neurons of the medial septum and an amelioration of the altered microglial morphology in neocortical areas. Furthermore, hNGFp rescued the expression levels of important molecules involved in neuroimmune communication, such as fractalkine (also known as CX3CL1).^28^ Conclusively, our work supports the broad neuroprotective actions of hNGFp and the use of the nasally-delivered neurotrophin-derivative hNGFp as a prospective therapy for Rett syndrome.

## Material and methods

### Animals

Mecp2^tm1.1Bird/J^ and control female mice were purchased from The Jackson Laboratories. Genotyping of *MeCP2*^+/−^ was performed by PCR analysis of tail DNA (9875: AAA TTG GGT TAC ACC GCT GA, Common; 9877: CCA CCT AGC CTG CCT GTA CT, Mutant Reverse; 7172: CTG TAT CCT TGG GTC AAG CTG, WT Reverse). All experiments were conducted according to the ARRIVE guidelines (Animal Research: Reporting *In Vivo* Experiments). Animals were randomized and coded so that the persons carrying out behavioural analysis, tissue processing and statistical analysis were blind to the treatment. Randomization was carried out using the Research Randomizer Program online (www.randomizer.org). The GPower program was used to calculate a *priori* the sample size. Power, alpha and effect size were set at 80%, 0.05 and 0.44, respectively. The number of subjects in the experiments is reported in the figure legends. All experiments with mice were performed according to the national and international laws for laboratory animal welfare and experimentation (EU directive n. 2010/63/EU and Italian DL n. 26 04/03/2014). Mice were kept under a 12-h dark to light cycle, with food and water *ad libitum*.

### Intranasal treatment with hNGFp

Human NGF painless was administered intranasally at the dose of 0.54 μg/kg, as this is the highest dose that did not induce pain in the orofacial region in Capsoni *et al.*^23^ The peptide was diluted in 1 M PBS (137 mM NaCl, 2.7 mM KCl, 10 mM Na_2_HPO_4_, 1.8 mM K_2_HPO_4_ pH 7.4) and administered intranasally to wild-type and *MeCP2*^+/−^ mice, 3 µl at a time, alternating the nostrils, with a lapse of 2 min between each administration, for a total of 14 times. As a control, female wild-type and *MeCP2*^+/−^ mice were treated with PBS. The frequency of administration for intranasal delivery was three times per week (every 2 days). Two groups of treatments were performed. The first one started at 2 months of age and lasted until mice underwent humane sacrifice. The second treatment started when hindlimb clasping was at score 1 (see below), i.e. around 3 months of age, and lasted 1 month.

### Behaviour scoring and tests

The parameters were monitored by a blinded observer and scoring was performed as described in Guy *et al*.^29^

#### Immobility

The mouse was placed on a bench and observed and scored: 0 = as wild-type; 1 = decreased movement when compared to wild-type: extended freezing period when first placed on bench and longer periods spent immobile; and 2 = no movement when first placed on the bench, mouse can move if prodded.

#### Gait

The mouse was placed on a bench and observed and scored: 0 = as wild-type; 1 = hind legs are spread wider than wild-type with decreased pelvic elevation (‘waddling’ gait); and 2 = severe abnormalities: tremor when the mouse lifts its feet, walks backwards or ‘bunny hops’ by lifting both hind feet at once.

#### Hindlimb clasping

The mouse was observed when suspended by holding the base of the tail, and scored: 0 = legs splayed outwards; 1 = hindlimbs drawn towards each other (without touching) or one leg pulled into the body; and 2 = both legs drawn tightly so that they touch each other or the body.

#### Tremor

The mouse was examined while standing on the palm of the hand for 1 min. 0 = no tremor; 1 = intermittent weak tremor; 2 = continuous tremor or intermittent strong tremor.

#### Breathing

Movement of flanks was examined while the animal was standing still and scored: 0 = normal breathing; 1 = time spans of normal breathing interspersed with short periods of fast breathing or with pauses in breathing; and 2 = irregular breathing (gasping or panting).

#### General conditions

Body weight, presence of alopecia, and facies according to the mouse Grimace scale were recorded.

#### Aggregated score

Sum of scores were recorded for immobility, gait, hindlimb clasping, tremor, general conditions and breathing parameters.

#### Beam test

The beam test measures motor coordination. Mice were left to walk on a 60 cm long beam. Distance travelled and a sensorimotor score were recorded. The score was assessed as follows: 0 = could not walk and fell off the beam; 1 = could not walk but hung onto the beam; 2 = walked but fell off the beam; 3 = walked <10 cm; and 4 = walked >10 cm. Three trials per mouse were conducted and their score was averaged. Adapted from Hayashi *et al.*^30^

#### Rotarod

Rotarod test measures motor coordination and motor learning. Animals were placed on the motorized rod (30 mm in diameter) in the chamber. The animal was first habituated for 1 min at a constant speed (4 rpm) and put back in the home cage. After 10 min, during the trial phase, the rotation speed gradually increased from 4 to 40 rpm over the course of 5 min. The time latency was recorded when the animal was unable to keep up with the increasing speed and fell.

### Immunohistochemical analysis

Mice received terminal anaesthesia (chloral hydrate, 10 mg/kg), they were perfused with PBS and the brains were dissected in three parts: the cranial portion containing the basal forebrain and one of the occipital poles were fixed in 4% paraformaldehyde/PBS and stored at 4°C until sectioning. The second occipital pole was frozen on dry ice and stored at −80°C until use.^31,32^

#### ChAT/NeuN/DAPI staining and counting

Coronal sections containing the medial septum were first blocked for 2 h at room temperature with 5% bovine serum albumin (BSA) + 0.3% Triton-X-100 in PBS, then they were incubated overnight at 4°C with goat anti-choline acetyltransferase (anti-ChAT 1:250, AB144P, Merck Millipore) and rabbit anti-NeuN (1:1000, ABN90; Sigma-Aldrich). The following day sections were incubated with the secondary antibodies (i) donkey anti-goat 488 (Alexa Fluor, ThermoFisher); and (ii) donkey anti-rabbit RhodamineX (706-295-148; Jackson ImmunoResearch) at a final concentration of 1:500 for 2 h at room temperature. The sections containing the medial septum were then mounted using Fluoroshield with DAPI (F6057; Sigma-Aldrich) and acquired with an Apotome (Zeiss) using a 20× objective in Tiles mode in order to view the entire medial septum. ChAT^+^ neurons, NeuN^+^ neurons and DAPI^+^ cells were automatically counted using a custom MATLAB script.

#### Iba1 staining and microglia 3D reconstruction

Coronal sections (40 µm) containing the occipital cortex were cut using a microtome (Leica Microsystems). After 2 h of blocking in 10% BSA + 0.3% Triton-X-100 in PBS, sections were incubated with a mix of primary antibodies in 1% BSA + 0.05%Triton-X-100 in PBS overnight at 4°C. Microglia were stained with rabbit anti-Iba1 1:500 (Wako, 019–19741). Sections were then incubated for 2 h at room temperature with the appropriate secondary antibody (Thermo Fisher Scientific; A-21428 diluted 1:500). Images were collected at a LSM 900 confocal microscope (Zeiss) using a Plan-Apochromat 63×, NA: 1.4 oil objective. *Z*-stacks of ∼25 μm were acquired (for a final voxel size of 0.1023810 µm × 0.1023810 µm × 0.68 μm). The Filament Tracer Tool of IMARIS software (Bitplane) was used to reconstruct microglia. For the density analysis, images were collected using a Plan-Apochromat 20×, NA:0.8. Tiles (single-plane) were acquired (for a final pixel size of 0.645 µm × 0.645 µm). The Cell Counter plug-in in ImageJ Fiji^33^ was used for the quantification.

### Measurement of inflammatory markers

Simultaneous detection of multiple cytokines was obtained using a mouse inflammation antibody array (RayBio® C-SeriesMouse Inflammation Antibody Array C1). Briefly, brain samples were homogenized in RIPA buffer (50 mM Tris/HCl, 150 mM NaCl, 1 mM EDTA, 1% Igepal, 0.5% sodium deoxycholate, 0.1% SDS, protease cocktail inhibitor) and protein content was determined using the Bradford method. Antibody arrays were incubated for 2 h at room temperature with blocking buffer. Protein extract (500 μg) was diluted in blocking buffer and incubated with the array overnight at 4°C. Then, antibody arrays were washed according to the manufacturer’s instructions and incubated for 3 h at room temperature with the biotinylated antibody cocktail solution. After washing, arrays were incubated with HRP-streptavidin for 2 h and developed using the detection buffer. Images were captured using the ChemiDoc detection system (Bio-Rad). Quantification was performed by measuring mean fluorescence in ImageJ Fiji,^33^ subtracting background and normalizing for the positive control spots, as suggested by the datasheet of the assay.

The analysis of fractalkine expression by western blot was performed on the intracellular fraction of brain extracts obtained as described before.^23^ Extracts were loaded on a 4–12% NU-PAGE bis-Tris pre-cast gels. Nitrocellulose membranes were incubated in a solution containing primary antibodies (anti-fractalkine 1:1000, GTX74237, GeneTex Inc.) were detected using anti-rabbit IgG conjugated with horseradish peroxidase (1:2000, #7074, Cell Signaling Technology). Blots were imaged with Chemidoc detection system (Bio-Rad). Densitometry analyses were performed using the NIH ImageJ 1.44p program.

### Primary cell cultures

#### Microglia

Primary microglial cells were derived from the brains of Mecp2^tm1.1Bird/J^ mice at P3–4 as previously described.^11^ Briefly, animals were decapitated and the brain was rapidly excised and placed into ice-cold PBS. Cortex was removed and digested for 5 min at 37°C in Dulbecco’s modified Eagle medium (DMEM/F12; Thermo Fisher Scientific) containing 0.1% of trypsin (Thermo Fisher Scientific). Tissue was transferred in culture medium containing 10% fetal bovine serum (FBS) and gently disrupted using a flame-polished Pasteur pipette. Following centrifugation at 4°C for 5 min at 1000 rpm, cells were resuspended and maintained in DMEM/F12 containing 1% penicillin/streptomycin, 1% GlutaMAX and 10% FBS in 5% CO_2_ pH 7.4 at 37°C. Microglia were separated from the mixed primary glial cultures by mild shaking, they were resuspended in DMEM/F12 with 1% penicillin/streptomycin, 1% GlutaMAX and 10% FBS and plated on the appropriate support 18 h before the experiments.

#### Neurons

Primary cortical neurons were prepared at P0 as previously described.^11^ Briefly, animals were decapitated, the brain was rapidly excised and placed into ice-cold Hanks buffered saline solution (HBSS; Thermo Fisher Scientific). Cortex was removed and digested for 15 min at 37°C in HBSS containing 0.1% of trypsin (Thermo Fisher Scientific). Tissue was transferred in culture medium containing 10% FBS and gently disrupted using a flame-polished Pasteur pipette. Following centrifugation at 4°C for 8 min at 800 rpm, cells were resuspended in fresh DMEM containing 1% GlutaMAX, 10% FBS, 2% B27 supplement (Gibco), 6 mg/ml glucose, 12.5 mM glutamate, 10 µg/ml gentamicin (Gibco) and plated (150 000 cells/coverslip) after proper poly-D-lysine coating (Sigma-Aldrich). Cells were kept at 37°C in 5% CO_2_. After 12–24 h, medium was replaced with Neurobasal A medium (Thermo Fisher Scientific) containing 2% of B27 supplement, 2.5 mM GlutaMAX and 10 µg/ml Gentamicin. On the second day, 2.5 mM AraC (Sigma-Aldrich) was added to the medium.

#### Neuron-microglia co-culture

At DIV (days *in vitro*) 17–19 for neurons, primary microglia were seeded onto cultured cortical neurons (1 × 10^5^ cells/well). The culture was maintained in Neurobasal-A supplemented with 2% B27, 2 mM L-glutamine and 10 µg/ml gentamicin and used after 24 h for experiments. Co-cultures were treated with 100 ng/ml hNGFp for 3 h, fixed in 2% PFA and 5% sucrose for 10 min, washed in PBS and blocked for 1 h at room temperature in 1% BSA. Incubation with primary antibody was performed at the following concentrations: guinea pig anti-Homer1bc 1:500 (Synaptic System; #160023), rabbit anti-Iba1 1:500 (Wako; #019-19741). Images were collected at an LSM 900 confocal microscope (Zeiss) using a Plan-Apochromat 63×, NA:1.4 oil objective. *Z*-stacks of 3 µm in depth were acquired (for a final voxel size: 0.0990358 µm × 0.0990358 µm × 0.14 µm), using Airyscan super-resolution. Images first went through image subtraction and thresholding, then Homer1bc^+^ puncta and dendritic length were quantified using Fiji by a blinded operator.^33^

### Statistical analysis

Data are displayed as individual dots and mean ± standard error of the mean (SEM). Differences between multiple groups were analysed either by one-way ANOVA or a two-way ANOVA when having more than one variable with appropriate *post hoc* tests defined in the figure legend. Normality was assessed using the Anderson-Darling test. Comparisons between two groups following a normal distribution were analysed using an unpaired *t*-test (two-tail distribution). Statistical analysis was performed using GraphPad Prism 8.0. Differences were considered to be significant if *P < 0.05; **P < 0.01; ***P < 0.001; and ****P < 0.0001.

The correlation data, principal component analysis (PCA) and Pearson coefficients, and related figures were obtained with Python scripts using the python libraries *sklearn and scipy*.

## Results

### A long-term treatment with hNGFp ameliorates behaviour and increases lifespan of *MeCP2*^+/−^ mice

**Figure 1:**
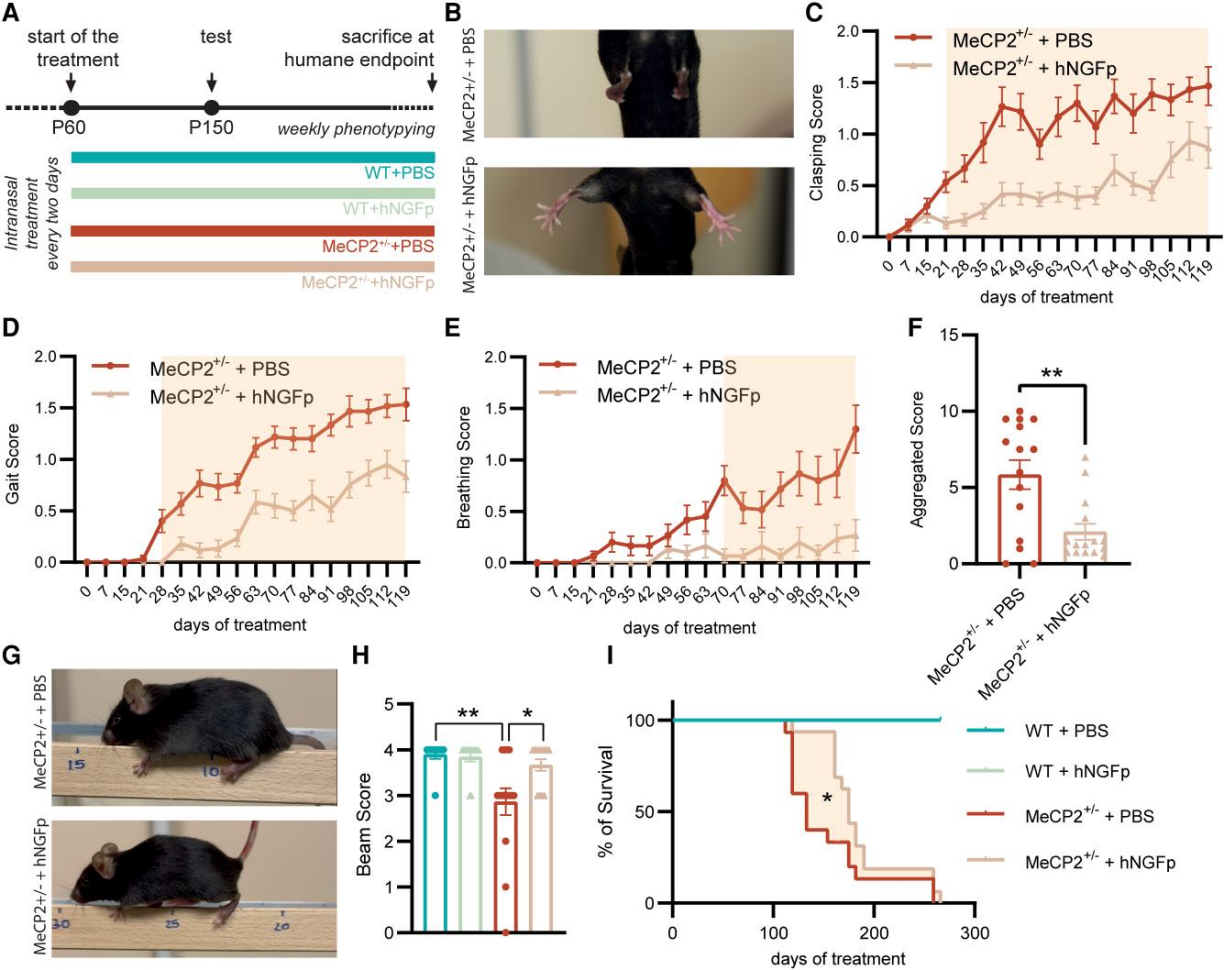
**Intranasal delivery of hNGFp ameliorates behavioural deficits and increases lifespan in MeCP2^+/−^ mice.** (**A**) Timeline: mice started treatment at 2 months of age and underwent weekly phenotyping until sacrifice. Motor performance was evaluated at P150. (**B**) Representative images of hindlimb clasping in vehicle (PBS) and hNGFp-treated MeCP2^+/−^ animals. (**C**–**E**) Plots indicating the (**C**) clasping, (**D**) gait and (**E**) breathing score measured once per week from the beginning of the treatment up until the humane end point [Clasping: two-way repeated measures ANOVA and Sidak’s multiple comparisons test; interaction: *F*(17,476) = 3.327, *P* < 0.0001; Gait: two-way repeated measures ANOVA and Sidak’s multiple comparisons test; interaction: *F*(17,476) = 4.842, *P* < 0.0001; mean ± SEM; Breathing: two-way repeated measures ANOVA and Sidak’s multiple comparisons test; interaction: *F*(17,476) = 4.405, *P* < 0.0001); orange box indicates significance in the multiple comparison test]. (**F**) Aggregated score calculated at Day 119 after the beginning of the treatment (unpaired two-tailed *t*-test; *P* = 0.0019; mean ± SEM and individual values). (**G** and **H**) Representative images and quantification of the score during a beam walk test [two-way ANOVA; Interaction: *F*(1,49) = 4.867; *P* = 0.0321; Tukey’s multiple comparisons test: WT + PBS versus MeCP2^+/−^ + PBS, *P* = 0.0035; MeCP2^+/−^ + PBS versus MeCP2^+/−^ + hNGFp; *P* = 0.0142; mean ± SEM and individual values] (*n* = 13–15 per group). (**I**) Kaplan-Meier survival analysis. MeCP2^+/−^ + PBS versus MeCP2^+/−^ + hNGFp; *P* = 0.01, Gehan-Breslow-Wilcoxon test. hNGFp = human nerve growth factor painless.

To evaluate the efficacy of hNGFp as a therapeutic agent for Rett syndrome, 2-month-old female *MeCP2*^+/−^ mice (i.e. just before the appearance of behavioural deficits) were intranasally treated three times per week with hNGFp or vehicle (PBS) up to a humane sacrifice end point, to determine survival (Fig. 1A). A thorough behavioural testing was performed on a weekly basis throughout the treatment period. First, we found that long-term hNGFp nasal treatment significantly ameliorated a wide range of behavioural parameters (hindlimb clasping, gait, breathing, tremor, immobility, general conditions) as well as the overall aggregated phenotypic score (see the ‘Materials and methods’ section, Fig. 1B–F and Supplementary Fig. 1C and D). At 5 months of age (∼ P150), mice were been tested for motor coordination and performance using a beam walk test, in which the animals were left to walk on a 60 cm long beam until they managed to cross it or fell off. Interestingly, hNGFp rescued both the distance travelled on the beam and the overall beam test score (see the ‘Materials and methods’ section), although they still spent more time completing the task compared to wild-type controls (Fig. 1G and H and Supplementary Fig. 1E and F).

As far as safety-related end points are concerned, no significant effect of hNGFp treatment on body or brain weight was detected after such a long treatment (∼37 weeks) (Supplementary Fig. 1A and B). Second, in line with the remarkable behavioural improvement observed, we found that hNGFp treatment significantly increased the lifespan of *MeCP2*^+/−^ animals (Fig. 1I), with an increase in median survival of 30%.

In conclusion, life-long treatment of *MeCP2*^+/−^ mice with nasally delivered hNGFp, starting at 2 months of age, ameliorates in a very significant way behavioural parameters and increases survival, suggesting a strong neuroprotective action of potentially high clinical relevance.

### Effects of a short hNGFp intranasal treatment in *MeCP2*^+/−^ mice

Having established that long-term nasal delivery of hNGFp ameliorates behavioural deficits and increases lifespan of *MeCP2*^+/−^ female mice, it was of interest to gain some insight into the molecular and cellular underpinning of the likely neuroprotective actions of hNGFp in this mouse model.

**Figure 2:**
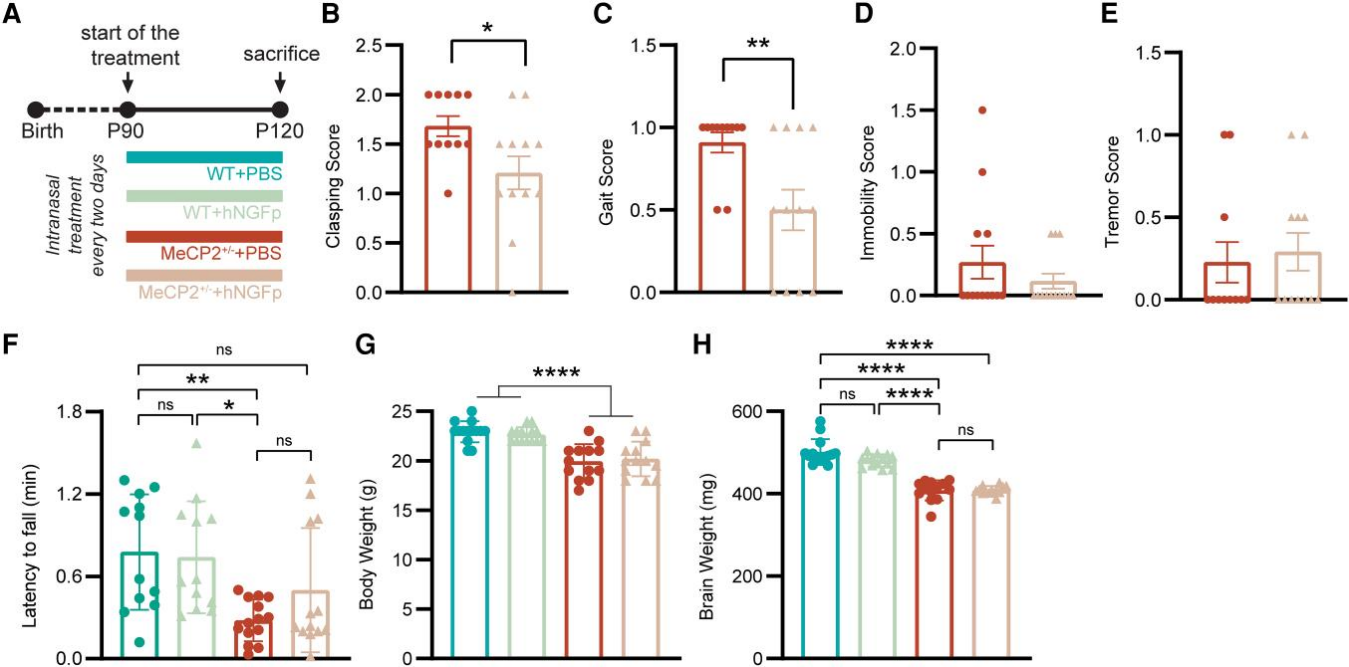
**Effect of a 1-month hNGFp treatment.** (**A**) Timeline of the experiment. (**B**–**E**) Scatter plot with bars of the behavioural score recorded the day before sacrificing and collecting the tissues of PBS- and hNGFp-treated MeCP2^+/−^ animals. (**B**) Clasping: two-tailed *t*-test; *P* = 0.0282. (**C**) Gait: two-tailed *t*-test; *P* = 0.0087. (**D** and **E**) Immobility and tremor: two-tailed *t*-test. Mean ± SEM and individual values are reported in all scatter plots with bar. (**F**) Latency to fall expressed in minutes in a rotarod test [two-way ANOVA; column effect (genotype): *P* = 0.001; Interaction: *P* = 0.2; Tukey’s multiple comparisons test: WT + PBS versus MeCP2^+/−^ + PBS: *P* = 0.0085; MeCP2^+/−^ + PBS versus WT + hNGFp: *P* = 0.0179]. (**G**) Body weight of all groups recorded right before sacrifice [two-way ANOVA; Genotype: *F*(1,50) = 52.35, *P* < 0.0001; mean ± SEM]. (**H**) Brain weight was recorded for all animals at the time of death [two-way ANOVA: Interaction: *F*(1,50) = 3.257; *P* = 0.0772; Tukey’s multiple comparisons test: WT + PBS versus MeCP2^+/−^ + PBS: *P* < 0.0001; WT + PBS versus MeCP2^+/−^ + hNGFp: *P* = 0.0004; MeCP2^+/−^ + PBS versus WT + hNGFp: *P* < 0.0001; WT + hNGFp versus MeCP2^+/−^ + hNGFp: *P* < 0.0001] (*n* = 12–14 per group). hNGFp = human nerve growth factor painless; WT = wild-type.

We sought therefore to investigate the effect of a shorter intranasal hNGFp treatment, started after the onset of behavioural deficits, in a therapeutic setting. To this aim, a second cohort of wild-type and *MeCP2*^+/−^ female mice was treated three times per week consecutively for 1 month, starting when hindlimb clasping was at score 1 (see below), i.e. ∼3 months of age (Fig. 2A).

#### Short treatment with intranasal hNGFp partially improves Rett-relevant behaviours

This short 1-month treatment was sufficient to improve some of the analysed behavioural parameters in *MeCP2*^+/−^ mice, in particular, gait and clasping score (Fig. 2B and C). On the other hand, immobility and tremor were not improved (Fig. 2D and E), nor was the motor performance on the rotarod test (Fig. 2F). No effect of the treatment was detected on body and brain weight (Fig. 2G and H), consistent with the experimental group in the long-term hNGFp treatment (Supplementary Fig. 1A and B).

#### Rescue of cholinergic deficits in *MeCP2*^+/−^ mice by short-term nasal delivery of hNGFp

**Figure 3:**
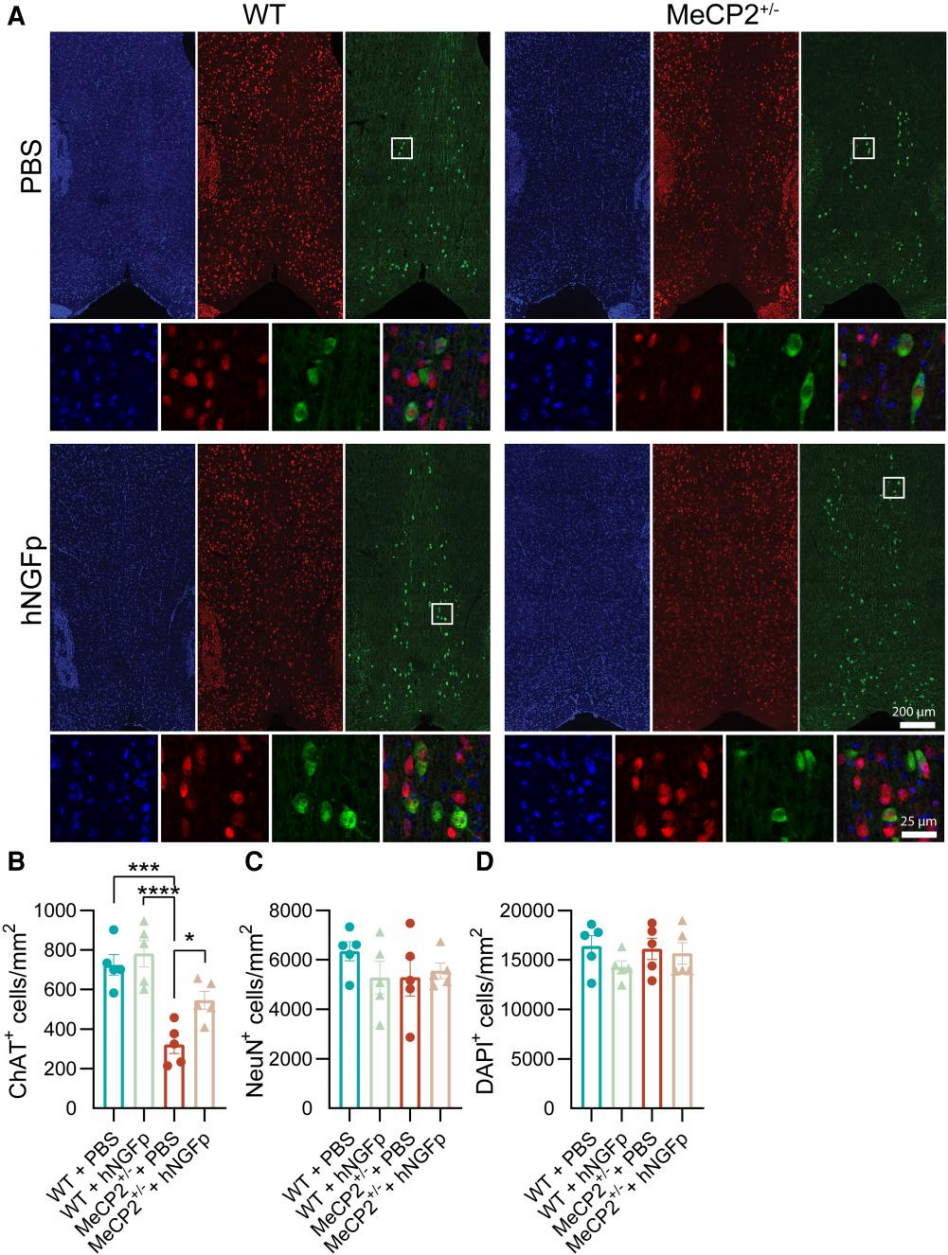
**The density of ChAT^+^ neurons in the medial septum is rescued by hNGFp treatment.** (**A**) Representative figures of DAPI, ChAT and NeuN fluorescent staining in the medial septum of WT + PBS, WT + hNGFp, MeCP2^+/−^ + PBS and MeCP2^+/−^ + hNGFp animals and their magnifications (scale bars are indicated in the panels). (**B**–**D**) Bar plots indicating the density of (**B**) ChAT^+^ cells, (**C**) NeuN^+^ cells and (**D**) DAPI (two-way ANOVA; Tukey’s multiple comparisons test for all plots) (*n* = 5 animals per group). (**B**) Density of ChAT^+^ cells [Interaction: *F*(1,16) = 2.461: *P* = 0.1363; effect of treatment: *F*(1,16) = 6.992: *P* = 0.0177; effect of genotype: *F*(1,16) = 36.06: *P* < 0.0001; WT + PBS versus MeCP2^+/−^ + PBS: *P* = 0.0003; MeCP2^+/−^ + PBS versus MeCP2^+/−^ + hNGFp: *P* = 0.0398; MeCP2^+/−^ + PBS versus WT + hNGFp: *P* < 0.0001]. (**C**) Density of NeuN^+^ cells [Interaction: *F*(1,16) = 1.391: *P* = 0.2555; effect of treatment: *F*(1,16) = 0.5170: *P* = 0.48; effect of genotype: *F*(1,16) = 0.4681: *P* = 0.5036]. (**D**) Density of DAPI [Interaction: *F*(1,16) = 0.6765: *P* = 0.4229; effect of treatment: *F*(1,16) = 1.691: *P* = 0.2119; effect of genotype: *F*(1,16) = 0.3096: *P* = 0.5856]. hNGFp = human nerve growth factor painless; WT = wild-type.

Given the Rett-related cholinergic deficits^10^ and the importance of NGF for the activity and survival of cholinergic neurons,^8,9^ we proceeded to analyse the effect of 1-month hNGFp treatment on the density of this population of neurons in the medial septum (Fig. 3A). First, we observed that density of ChAT^+^ cells was substantially lower in 4-month-old *MeCP2*^+/−^ animals (Fig. 3B). Interestingly, this was not accompanied by a reduction in NeuN^+^ cells or DAPI (Fig. 3C and D, respectively), suggesting a decrease in the expression of ChAT and not a loss of cholinergic neurons *per se*. This is consistent with reports indicating that loss of MeCP2 specifically in the basal forebrain decreases ChAT expression.^34^ Moreover, we found that treatment with hNGFp rescued ChAT expression in cholinergic neurons (Fig. 3B), consistent with previous results indicating that NGF can induce the expression of ChAT mRNA in this population of cells.^35^ In conclusion, our data reveal that *MeCP2*^+/−^ animals suffer from cholinergic deficits that can be fully rescued via the intranasal administration of hNGFp.

### Human NGF painless rescues cortical microglia morphology

Microglial cells greatly contribute to brain development: they assist in properly wiring excitatory and inhibitory circuits, they can affect neuronal maturation in neurogenic niches and they work in unison with other glial cells to maintain homeostasis in the ever-changing developing brain.^36,37^ These cells are thus believed to be involved in the pathogenesis of many neurodevelopmental disorders, including Rett syndrome.^38^ In fact, morphological abnormalities in microglia have been identified in *MeCP2*-null mice.^16^

**Figure 4:**
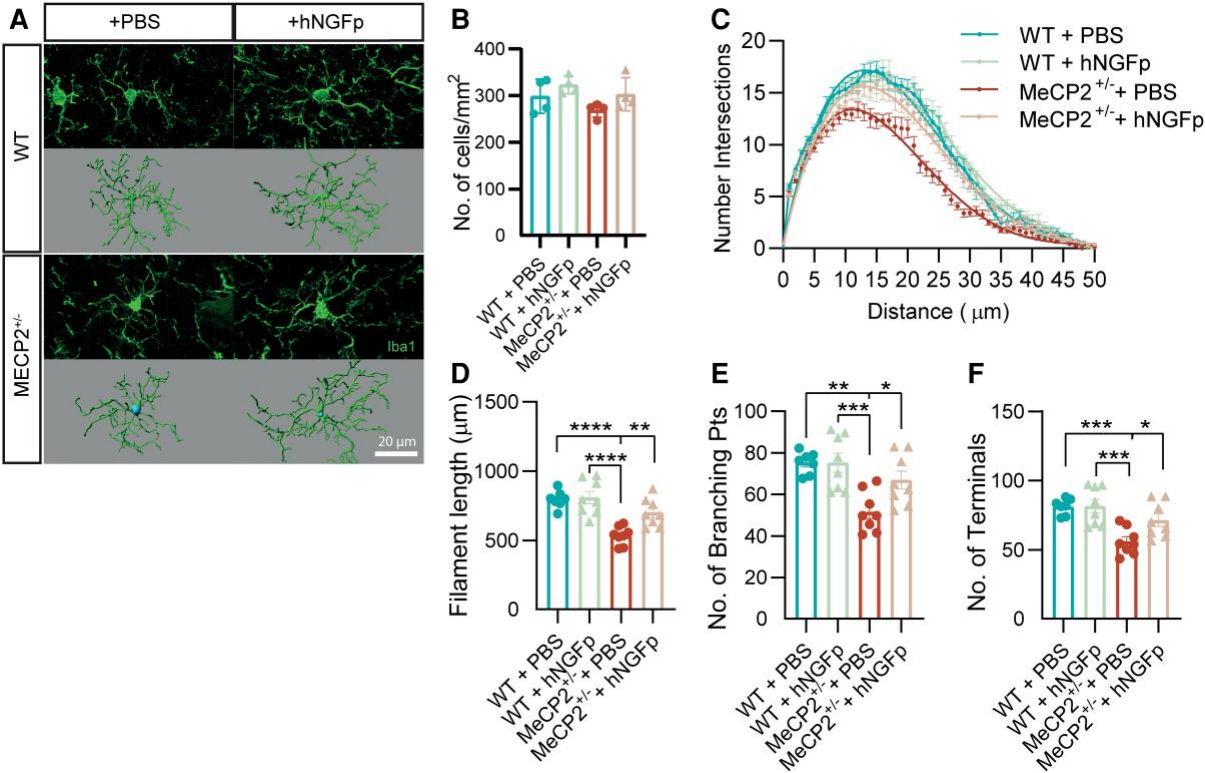
**Human NGF painless rescues cortical microglia morphology.** (**A**) Representative images of Iba1 immunofluorescence and IMARIS 3D reconstruction for each of the analysed groups. Scale bar = 20μm. (**B**) Scatter plot with bar representing the density of microglial cells in the cortex of all cohorts of animals. No significant difference was detected [two-way ANOVA, *F*(1,12) = 0.1165, *P* = 0.7388; *n* = 5 animals per group]. (**C**–**F**) Results from IMARIS 3D reconstruction (*n* = 3–5 cells per group; *n* = 8 animals per group). (**C**) 3D Sholl analysis [three-way ANOVA: Interaction: Genotype × Treatment: *F*(1,2128) = 54.54, *P* < 0.0001; Radius × Genotype Treatment, *F*(75,2128) = 1.377, *P* = 0.0191]. (**D**–**F**) Scatter plots with bars representing filament length, number of branching points and terminal points, respectively (two-way ANOVA, Tukey’s multiple comparisons test for all plots). (**D**) Filament length: *F*(1,27) = 5.237, *P* = 0.0302; WT + PBS versus MeCP2^+/−^ + PBS: *P* < 0.0001; WT + PBS versus MeCP2^+/−^ + hNGFp: *P* = 0.22; MeCP2^+/−^ + PBS versus MeCP2^+/−^ + hNGFp: *P* = 0.0072; MeCP2^+/−^ + PBS versus WT + hNGFp: *P* < 0.0001. (**E**) Number of branching points: *F*(1,27) = 3.973, *P* = 0,0.0564; WT + PBS versus MeCP2^+/−^ + PBS: *P* = 0.0011; WT + PBS versus MeCP2^+/−^ + hNGFp: *P* = 0.44; MeCP2^+/−^ + PBS versus MeCP2^+/−^ + hNGFp: *P* = 0.0370; MeCP2^+/−^ + PBS versus WT + hNGFp: *P* = 0.0007. (**F**) Number of terminal points: *F*(1,27) = 3.511, *P* = 0.0718; WT + PBS versus MeCP2^+/−^ + PBS: *P* = 0.0008; WT + PBS versus MeCP2^+/−^ + hNGFp: *P* = 0.34; MeCP2^+/−^ + PBS versus MeCP2^+/−^ + hNGFp: *P* = 0.046; MeCP2^+/−^ + PBS versus WT + hNGFp: *P* = 0.0005. Mean ± SEM and individual values are reported in all scatter plots with bar. hNGFp = human nerve growth factor painless; WT = wild-type.

As mentioned above, we and others have identified microglial cells as new putative targets of NGF in the brain.^25,26^ Thus, we asked whether the hNGFp treatment could influence *MeCP2*^+/−^ microglia. At first, we quantified cortical microglial density and found no difference between wild-type and *MeCP2*^+/−^ animals in all treatment groups (Fig. 4B). To further analyse these myeloid cells, we quantified a series of morphological parameters using the 3D morphometric analysis (Fig. 4A). Notably, the morphology of microglia is closely related to their functional state, as these cells are constantly surveying the surrounding environment and contact neurons with their ever-moving processes.^39-41^ Interestingly, using Sholl analysis, which relates a measure of the branching complexity of cells, we observed a reduction in arborization of microglia in *MeCP2*^+/−^ animals (Fig. 4C). Importantly, this morphological difference between wild-type and *MeCP2*^+/−^ mice was completely abolished by hNGFp treatment. To then understand more about these changes, we analysed the length and branching properties of microglial processes. We observed that hNGFp could revert a decrease in the overall length of microglial arborizations and in the number of branching and terminal points (Fig. 4D–F). In conclusion, our data indicate that microglial morphology is severely affected in *MeCP2*^+/−^ animals, suggesting a direct role of microglia in the pathogenic process in *MeCP2*^+/−^ mice. Importantly, hNGFp treatment determined a full rescue of these morphological abnormalities.

**Figure 5:**
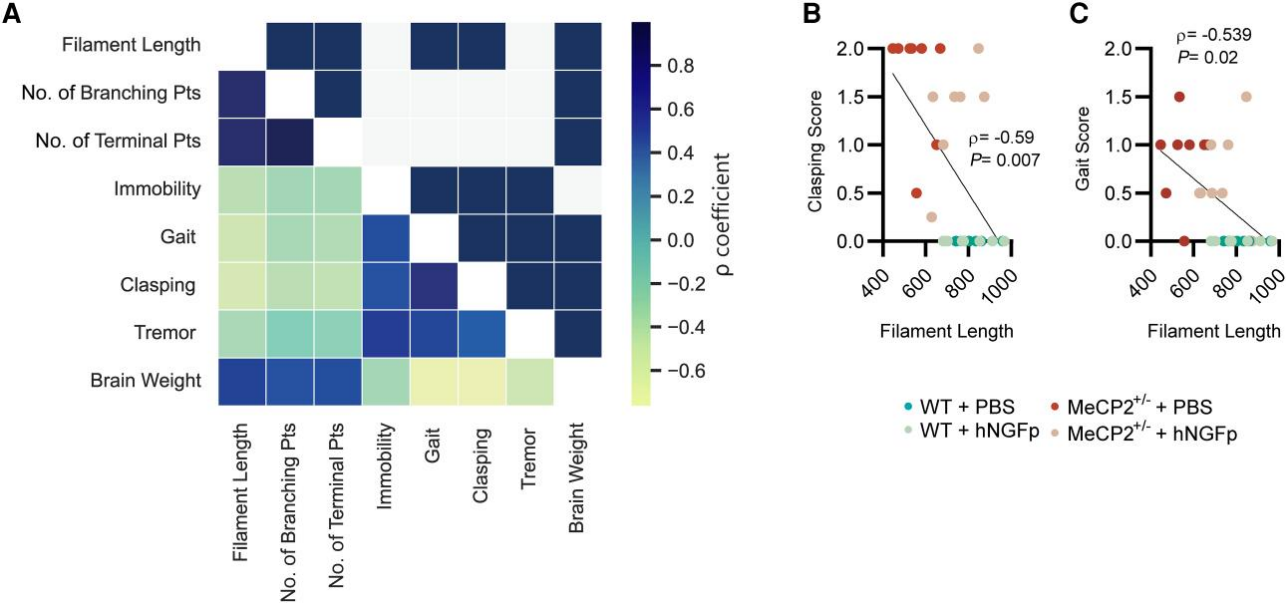
**Microglial morphological parameters are highly predictive of behavioural output.** (**A**) Heat map representing values from the correlation matrix (Pearson correlation) of all analysed parameters (*bottom* triangle), and the significance of each pairwise comparison (*top* triangle: blue box indicates *P* < 0.05 after Holm-Sidak multiple testing correction) (all groups were included in the analysis, *n* = 8 animals per group). (**B** and **C**) Detail on clasping and gait correlation with filament length Pearson coefficient and corrected *P*-values, respectively are reported in the figure. hNGFp = human nerve growth factor painless; WT = wild-type.

To further investigate the relevance of these data, we calculated the correlation between microglial morphological parameters and *MeCP2*^+/−^-relevant behavioural data, such as immobility, gait, hindlimb clasping and tremor. Significantly, *MeCP2*^+/−^-relevant behavioural scores were inversely correlated with microglial morphological parameters, suggesting that proper ramification of these cells is indicative of a good behavioural outcome (Fig. 5A). In particular, we found a significant negative correlation between microglial filament length and both gait and hindlimb clasping (Fig. 5B and C). Of interest, brain weight negatively correlated with behavioural parameters (the higher the brain weight, the better the phenotype) and positively correlated with microglial parameters (the bigger the brain, the more complex and ramified the microglia) (Supplementary Fig. 2). These correlations highly support the involvement of microglia in determining disease progression.

#### Human NGF painless modulates the protein levels of different cytokines

**Figure 6:**
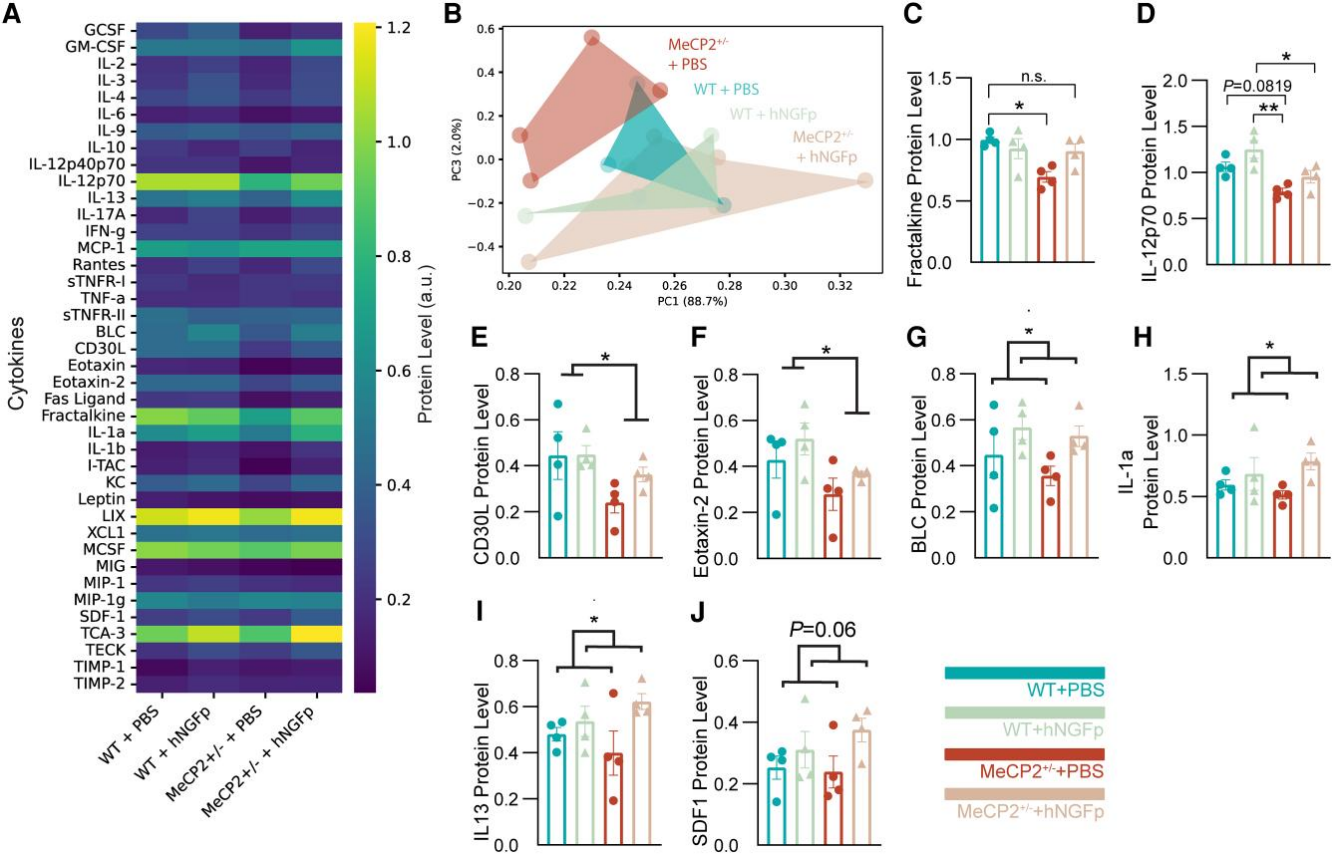
**Human NGF painless modulates the expression levels of disease-relevant cytokines in *MeCP2*^+/−^ mice.** (**A**) Heat map showing the protein levels of cytokines in WT + PBS, WT + hNGFp, *MeCP2*^+/−^ + PBS and *MeCP2*^+/−^ + hNGFp cohorts of mice, quantified using the RayBio® C-Series mouse inflammation antibody array. (**B**) PCA analysis showing the clustering of the different treatments. (**C**–**K**) Histogram plots showing the protein levels of selected cytokines from the array and the statistical analysis from the two-way ANOVA and Sidak’s multiple comparisons test. (**C**) Fractalkine: Interaction: *F*(1,12) = 6.155, *P* = 0.0289; effect of Genotype: *F*(1,12) = 8.145, *P* = 0.0145; WT + PBS versus *MeCP2*^+/−^ + PBS: *P* = 0.0159; MeCP2^+/−^ + PBS versus *MeCP2*^+/−^ + hNGFp: *P* = 0.0805. (**D**) IL-12p70: effect of Treatment: *F*(1,12) = 6.915, *P* = 0.022; effect of Genotype: *F*(1,12) = 18.11, *P* = 0.001; WT + PBS versus *MeCP2*^+/−^ + PBS: *P* = 0.0819; MeCP2^+/−^ + PBS versus WT + hNGFp: *P* = 0.0023; WT + hNGFp versus *MeCP2*^+/−^ + hNGFp: *P* = 0.0493. (**E**) CD30L: effect of Genotype: *F*(1,12) = 5.619, *P* = 0.0354. (**F**) Eotaxin-2: effect of Genotype: *F*(1,12) = 5.639, *P* = 0.0351. (**G**) BLC: effect of Treatment: *F*(1,12) = 5.154, *P* = 0.0424. (**H**) IL-1a: effect of Treatment: *F*(1,12) = 5.104, *P* = 0.0433. (**I**) IL-13: effect of Treatment: *F*(1,12) = 4.962, *P* = 0.0458. (**J**) SDF1: effect of Treatment: *F*(1,12) = 4.160, *P* = 0.064. (*n* = 4 per group, mean ± SEM and individual values are reported in all scatter plots with bar). hNGFp = human nerve growth factor painless; WT = wild-type.

In the past, hNGFp has been shown to act as an immunomodulator, affecting the expression of different cytokines that mediate its neuroprotective action in the brain.^23^ To better understand the activity of hNGFp on *MeCP2*^+/−^ animals, we analysed the expression levels of a variety of different cytokines in brain protein extracts from all treatment groups. Overall, the analysis revealed that the cytokine profile of PBS-treated *MeCP2*^+/−^ is strikingly different from that of the other cohorts (Fig. 6A). PCA indeed shows how PBS-treated *MeCP2*^+/−^ mice cluster differently to the largely overlapping cytokine profile of wild-type (WT) + PBS, WT + hNGFp and hNGFp-treated *MeCP2*^+/−^ mice (Fig. 6B and Supplementary Fig. 3). Looking at specific cytokines in the array, we could observe a specific rescue by hNGFp of Fractalkine protein levels (Fig. 6C), while IL-12p70 showed a significant modulation by genotype and treatment but no significant interaction (Fig. 6D). The analysis also revealed MeCP2-specific downregulated cytokines, such as CD30L and eotaxin-2 (Fig. 6E and F), suggesting that the immune profile in MeCP2 animals is compromised. Moreover, we could observe several cytokines that were similarly upregulated by hNGFp in both wild-type and *MeCP2*^+/−^ animals (Fig. 6G–J), indicating the action of the neurotrophin to be broad and not necessarily linked to the specific disease-associated context. To further corroborate these results, we validated the rescue of the fractalkine cytokine by western blot, finding similar results (and Supplementary Fig. 4). Overall, these data indicate that hNGFp can profoundly modulate the cytokine profile with significant disease-relevant consequences.

### Human NGF painless restores physiological levels of *MeCP2*^−/−^ dendritic spines in microglial-neuron co-culture

Neuroimmune communication is an essential mechanism that guarantees homeostasis in the brain. In particular, microglia are known to affect synapse number in co-culture with neurons, a process that is highly dependent on their inflammatory state and their cytokine expression profile.^11,42^ It has previously been reiterated that MeCP2 is a protein highly involved in synaptogenesis and, to add to that hypothesis, synaptic deficits as in spine numbers and motility have been described in murine models of Rett syndrome.^43,44^ We thus tested whether and how microglial cells could contribute to the synaptic deficits in an *in vitro* microglia-neuron co-culture model. To do so, we decided to co-culture healthy wild-type cortical neurons with microglia derived from *MeCP2*^−/−^ animals, in order to avoid the variability induced by the cellular mosaicism due to random X-inactivation in *MeCP2*^+/−^ animals.^45^

**Figure 7:**
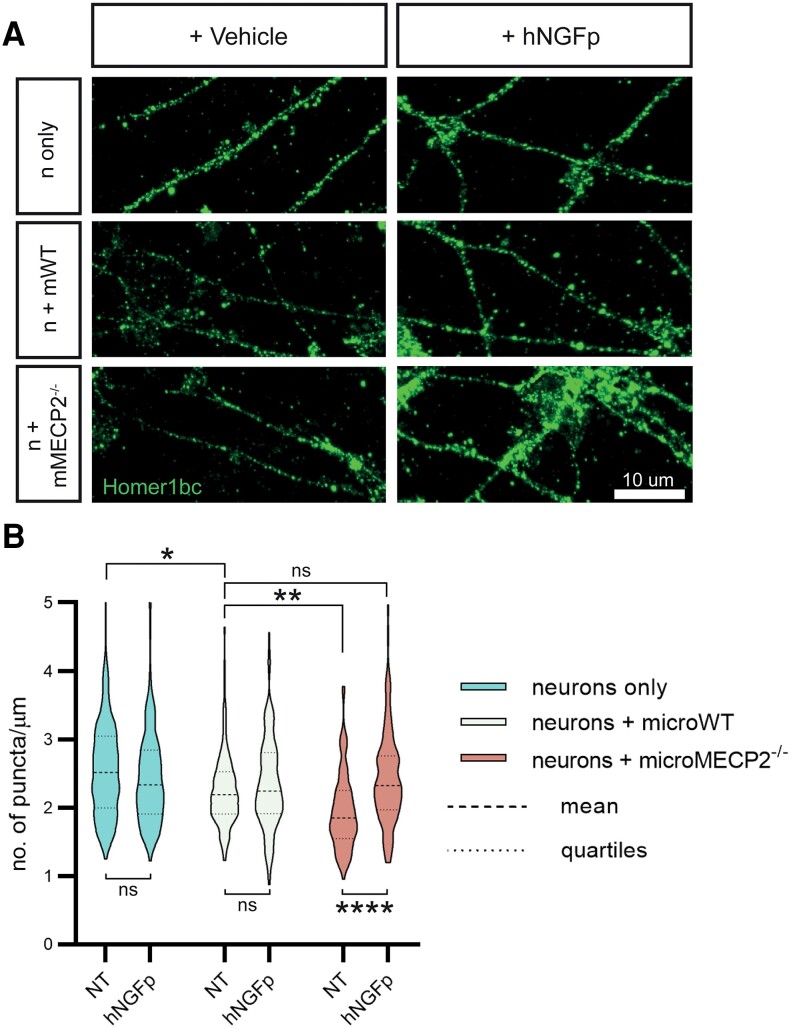
**Human NGF painless rescues microglia neuroprotective activity towards spines.** (**A**) Representative images of Homer1bc staining for postsynaptic puncta in cortical neurons cocultured with microglia. The experimental conditions are: neurons only (n only), neurons + microglia WT (n + mWT), neurons + microglia from MeCP2^−/−^ mice (n + mMECP2^−/−^); cultures are either treated with 100 ng/ml of hNGFp or vehicle (NT = DMEMF12) for 3 h. Scale bar = 10 µm. (**B**) Violin plots of all quantified puncta densities in all conditions [three replicates, number of dendrites = 120 per group; two-way ANOVA; Interaction: *F*(2,624) = 11.48; *P* < 0.0001; Tukey’s multiple comparisons test: neurons only + NT versus neurons + microglia WT + NT: *P* = 0,0158; neurons + microglia MeCP2^−/−^ + NT versus neurons + microglia MeCP2^−/−^ + hNGFp: *P* < 0.0001; neurons + microglia WT + NT versus neurons + microglia MeCP2^−/−^ + NT: *P* = 0.0069]. hNGFp = human nerve growth factor painless; NT = non-treated/vehicle; WT = wild-type.

We then quantified synaptic density on neurons in response to the presence of wild-type or *MeCP2*^−/−^ microglia. First, we found a decrease in Homer1bc^+^ puncta in cortical neurons cultured with wild-type microglia (Fig. 7A and B), compared to pure neuronal cultures (neurons only), confirming previously published results.^11,42^ This reflects physiological ongoing synaptic pruning activity by microglia, under cultured conditions. Moreover, we report that co-culturing neurons with *MeCP2*^−/−^ microglia further decreases spine density, suggesting that, as hypothesized, microglia can indeed contribute to synaptic deficits in this Rett syndrome model. Then, we proceeded to understand whether hNGFp treatment could affect this deleterious behaviour of *MeCP2*^−/−^ microglia by administering the hNGFp neurotrophin to the co-culture system. Indeed, we observed that hNGFp can selectively counteract the adverse effect of *MeCP2*^−/−^ microglia on synaptic density (Fig. 7B). Importantly, hNGFp does not affect the physiological ongoing synaptic pruning activity by microglia, in line with previous results with NGF.^11^ In conclusion, our experiment highlights how MeCP2 mutations can directly impinge on microglial function, possibly affecting their ability to engage in proper neuroimmune communication. These deficits may have profound consequences on the structure and function of synaptic connections. It is therefore particularly significant that hNGFp treatment can re-establish a correct microglia-neuron homeostatic relationship.

## Discussion

Rett syndrome is a devastating disease with no cure in sight. The ubiquitously expressed mutated MeCP2 protein has dire effects on the CNS, where it affects a great variety of neuronal and non-neuronal populations.^2^ Therefore, there is a compelling need to find treatments that can have a brain-wide effect. In this work, we propose a therapeutic strategy for Rett syndrome, based on the non-invasive intranasal delivery of hNGFp. The hNGFp neurotrophin variant is endowed with a dual beneficial capacity, a direct trophic activity on target neuronal populations (cholinergic neurons) and a broad indirect neuroprotective activity, via microglia.^11,23,25^ As far as the delivery route is concerned, in previous work we have provided extensive evidence that nasal delivery is an effective route to deliver NGF and hNGFp to the brain, achieving a wide biodistribution across different brain regions, including, but not limited to, the cortex and the hippocampus.^23^ It is therefore of interest to test if the expected broad neuroprotective actions by nasally delivered hNGFp are effective in a well established mouse model for Rett syndrome, in which both cholinergic and microglial populations have been identified as important players.^10,14-16^

### Intranasal hNGFp is effective both in the pre- and post-symptomatic disease phases

Rett syndrome is an X-linked pathology and, as such, it primarily affects females. Since the *MeCP2* gene undergoes X-inactivation, patients are usually female heterozygotes, displaying variable cellular mosaicism for normal and mutant MeCP2.^2^ Rare cases of hemizygous male patients for mutant *MeCP2* have been reported but they rarely survive.^2^ In mice, *MeCP2* loss-of-function is less severe: male hemizygous null mice not only survive until adulthood but they also have been the most commonly studied model system for Rett syndrome, despite the fact that heterozygous female mice better recapitulate the human disease. In our study, we have thus specifically decided to evaluate the effect of an intranasal treatment with hNGFp on *MeCP2*^+/−^ female mice. In particular, (i) we report that a life-long treatment with hNGFp—starting during the presymptomatic phase of disease ∼2 months of age—ameliorates behavioural parameters and promotes survival in *MeCP2*^+/−^ female mice (Fig. 1). The results of this study showed a very significant and comprehensive behavioural improvement, accompanied by a 30% increase of the lifespan. Moreover, (ii) a 1-month treatment with the hNGFp neurotrophin beginning after symptom manifestation similarly improves Rett-relevant behaviour, albeit to a lower degree, most likely due to the rather short treatment duration (Fig. 2). This shorter trial allowed us to evaluate some of the molecular and cellular underpinnings of hNGFp treatment, in particular with respect to the known NGF-target cells in the brain, namely cholinergic neurons of the basal forebrain and microglia.

### Human NGF painless treatment positively affects cholinergic neurons

Cholinergic neurons in the basal forebrain are a well known target of NGF in the CNS.^8,9^ Therefore, we investigated whether and how this population of cells is involved in the beneficial effects of hNGFp treatment in our cohorts of mice. First, we observed a reduction in the number of ChAT^+^ cholinergic neurons, but not in the overall number of NeuN^+^ or DAPI^+^ cells, within the medial septum of 4-month-old *MeCP2*^+/−^ female mice. This observation likely suggests a loss of ChAT expression in *MeCP2*^+/−^ animals rather than an actual loss on neurons. This results nonetheless in a functionally reduced cholinergic drive in *MeCP2*^+/−^ mice. This is consistent with previous reports indicating that knocking out MeCP2 specifically in this population of neurons severely decreases the expression of the enzyme ChAT, thus impairing cholinergic neuronal function.^34^ Of note, we observed that hNGFp treatment could rescue such deficits (Fig. 3). This result is particularly relevant for the pathological and clinical phenotype of Rett syndrome. Indeed, loss of MeCP2 protein, specifically in cholinergic neurons, has been shown to be a strong driver of Rett-like phenotypes, such as learning deficits^46^ and seizure susceptibility.^34^ Moreover, decreased levels of acetylcholine have been reported in multiple brain regions of female *MeCP2*-deficient gene knockout rats,^47^ suggesting how cholinergic neuron activity might be substantially decreased in Rett syndrome models. Taken together, these findings suggest that MeCP2 is critical for the normal development, phenotypic maintenance and function of cholinergic neurons and, as a consequence, MeCP2-driven dysfunction of cholinergic neurons can contribute to numerous clinically relevant phenotypic aspects of Rett syndrome. The ability of hNGFp to rescue ChAT expression is thus of the utmost importance, as it might have beneficial effects on the cognitive impairment of patients with Rett syndrome. The result also provides a further and clear demonstration of the effective engagement of the nasally delivered hNGFp onto the cholinergic neurons of the medial septum.

### Human NGF painless affects the physiological functions of the brain immune system

We have also investigated the effect of hNGFp treatment on another cellular target of hNGFp in the brain, namely microglia cells.^11,23-26^ We show that the nasal hNGFp treatment positively affects the altered morphology of microglial cells, fully restoring their arborization parameters to those observed in the brains of wild-type mice (Fig. 4). We also report that morphological parameters of microglia from *MeCP2*^+/−^ mice are indicative of a good behavioural outcome, supporting the fact that these cells can be important contributors to the pathogenesis of the disease (Fig. 5).

The activity of hNGFp on the brain immune system was also determined by comparing the cytokine profile in all treatment groups, showing that the cytokine profile of hNGFp-treated *MeCP2*^+/−^ mice is different from that in *MeCP2*^+/−^ mice and largely overlapping with the cytokine profile of PBS and hNGFp-treated wild-type mice (Fig. 6B and Supplementary Fig. 3). Since proper communication between neurons and microglia heavily relies on the secretion of cytokines, changes in their levels can considerably affect neuronal activity and function.^48^ If we compare our cytokine analysis to existing microglia-specific RNA-seq data from 24-week-old *MeCP2*^+/−^ female mice published by Zhao *et al*.,^15^ we can observe that both IL12 and CD30L are specifically decreased in microglia from *MeCP2*^+/−^ female mice in their dataset. Our data show a similar trend (Fig. 6D and F) that can be partially rescued by hNGFp, suggesting a direct effect of the treatment on microglia for these two specific cytokines. Moreover, in our dataset, we observe a negative modulation of a known neuronal-released cytokine, fractalkine (also known as CX3CL1), in *MeCP2*^+/−^ naïve mice, which can be rescued by hNGFp (Fig. 6C and Supplementary Fig. 4). This suggests that hNGFp can affect the expression of important cytokines that are relevant both for microglia and neurons in *MeCP2*^+/−^ female mice. The effect of hNGFp on fractalkine is particularly relevant, as its interaction with the microglial chemokine receptor CX3CR1 is required for neuron-to-microglia communication. For instance, CX3CR1\CX3CL1 signalling is known to be particularly important for synaptic pruning by microglia during brain development.^49^ Moreover, rare genetic variants of CX3CR1 have been associated with schizophrenia and autism spectrum disorders^50^ and disruption of CX3CL1\CX3CR1 signalling has been previously reported in a *MeCP2* deficiency model.^51^ Our results thus independently confirm this deficiency and offer a therapeutic molecule capable of restoring this crucial signalling interaction between neurons and microglia, which is also disease-validated and, hence, potentially clinically very relevant.

To better dissect the role of microglia in MeCP2-driven pathogenesis, we have co-cultured neurons with microglia from wild-type and *MeCP2*^−/−^ mice (Fig. 7). Interestingly, we could observe a selective decrease in spine density in neurons co-cultured with *MeCP2*^−/−^ microglia, which was specifically rescued by hNGFp treatment. This result is particularly relevant as it indicates that *MeCP2*^−/−^ microglia can be deleterious on their own, possibly due to a different expression of cytokines and it is in line with previously published work that sees *MeCP2*-null microglia as a driver of synaptic deficits.^52^

Overall, the beneficial impact of hNGFp has the potential to improve neuronal physiology as a whole, either indirectly via microglia, which is steered to an overall broad neuroprotective phenotype, and directly, through specific actions on target neurons. Therefore, hNGFp might exert a positive influence also on neurons that are not its direct targets (that is, neurons that are not expressing TrkA) via its action on microglial cells.

In conclusion, these results, coupled with the broad biodistribution that can be achieved by intranasal delivery,^23^ make the nasal treatment with hNGFp a promising therapeutic avenue for genetic pathologies that affect different cell populations in the brain, such as Rett syndrome.

## Supplementary Material

awad282_Supplementary_Data

## Data Availability

The data that support the findings of this study are available from the corresponding author.
