# Supplementary material for: Reversal of neurological deficits by painless nerve growth factor in a mouse model of Rett syndrome
Source: Brain. 2023 Aug 26;147(1):122–34. doi: 10.1093/brain/awad282 (PMC10766238; doi:10.1093/brain/awad282)
Supplement: awad282_Supplementary_Data [file awad282_supplementary_data.pdf]

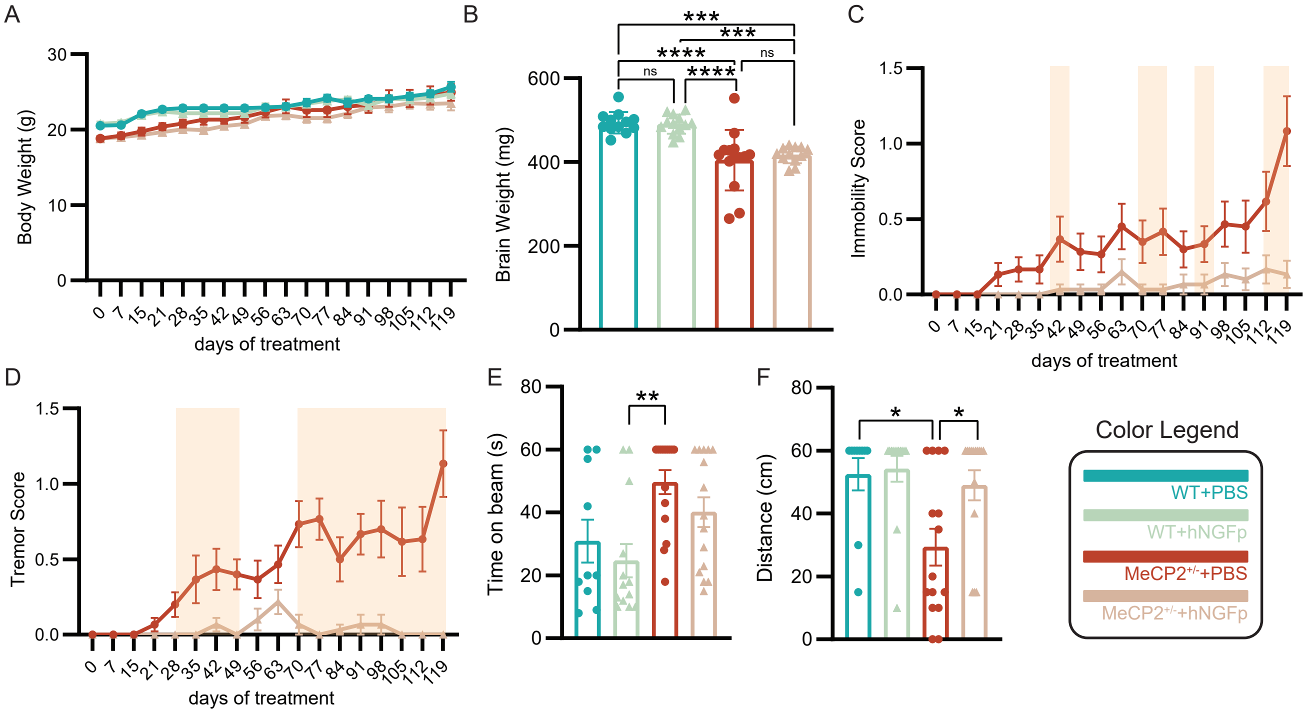

Supplementary Figure 1. Intranasal delivery of hNGFp ameliorates behavioral deficits and increases lifespan in MeCP2+/- mice. (A) Body weight of all groups recorded each week from beginning of treatment (2-WAY RM ANOVA ; Treatment: F (1, 51) = 1.197, p = 0.2790; Genotype: F (1, 51) = 6.992, p=0.0109; Mean ± SEM). (B) Brain weight was recorded for all animals at the time of death (2-WAY ANOVA - Interaction: F (1, 47) = 0.4591, p=0.5014; Sidak's multiple comparisons test: WT + PBS vs. MeCP2+/- + PBS: p<0.0001; WT + PBS vs. MeCP2+/- + hNGFp: p=0.0004; MeCP2+/- + PBS vs. WT + hNGFp: p<0.0001; WT + hNGFp vs. MeCP2+/- + hNGFp : p=0.0006). (C, D) Plots indicating the (C) Immobility and (D) Tremor score measured each week from the beginning of the treatment up until the beginning of death events (day of treatment 119); orange box indicates significance in the multiple comparison test (2-WAY RM ANOVA & Sidak's multiple comparisons test; Interaction significance details for: Immobility: F (17, 476) = 2.823, p=0.000; Tremor: F (17, 476) = 5.282, p<0.0001). (E, F) Plots showing the (E) time spent on the beam and (F) distance travelled during the Beam Walk Test for all groups (Time on beam (E): 2-WAY ANOVA & Sidak's multiple comparisons test; Interaction: F (1, 49) = 0.1078, p=0.7440; MeCP2+/- + PBS vs. WT + hNGFp p=0,0037; Distance (F): 2-WAY ANOVA & Sidak's multiple comparisons test; Interaction: F (1, 49) = 2.987, p=0.0902; MeCP2+/- + PBS vs. MeCP2+/- + hNGFp p=0,0338; MeCP2+/- + PBS vs. WT + hNGFp p=0,0055; WT + PBS vs. MeCP2+/- + PBS p=0,0222 (n=10-15 per group); Mean ± SEM and individual values are reported in all scatter plots with bars.

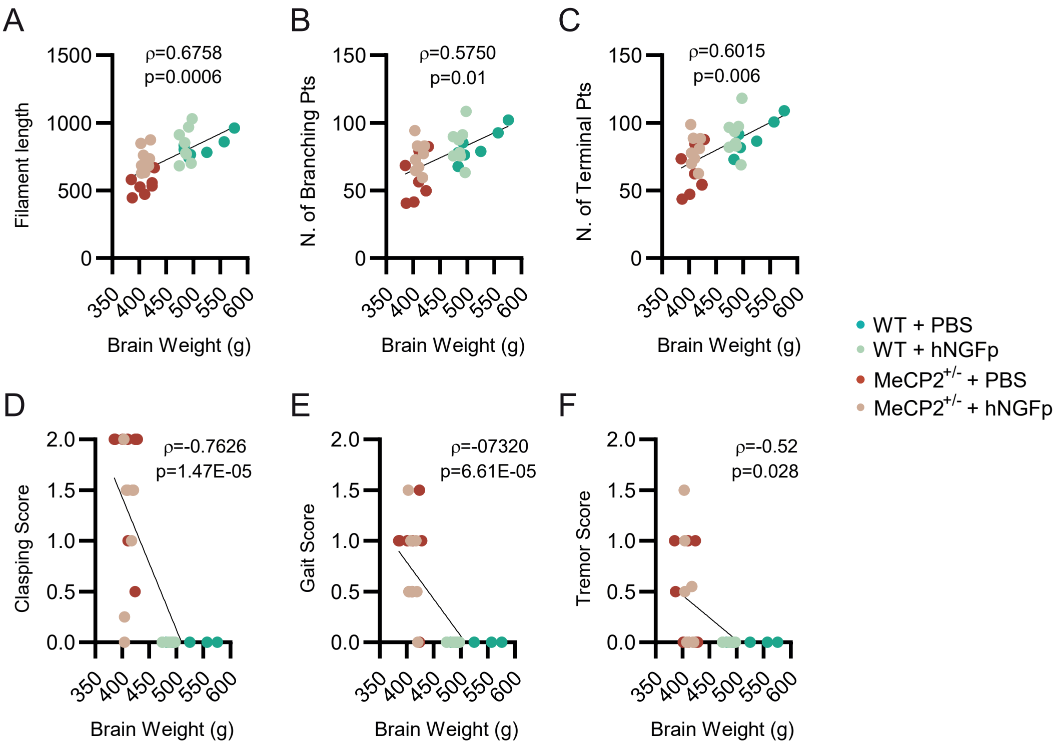

Supplementary Figure 2. Microglial morphological parameters are highly predictive of behavioral output. (A-C) Additional significant correlations from the correlation matrix in Figure 5 between Brain Weight and the microglial paramenters Filament Length (A), Number of Branching Points (B), Number of terminal Points (C) and the Behavioral parameters Clasping Score (D), Gait Score (E), Tremor Score (F); Pearson coefficients (ρ) and p-values are reported in the figures.

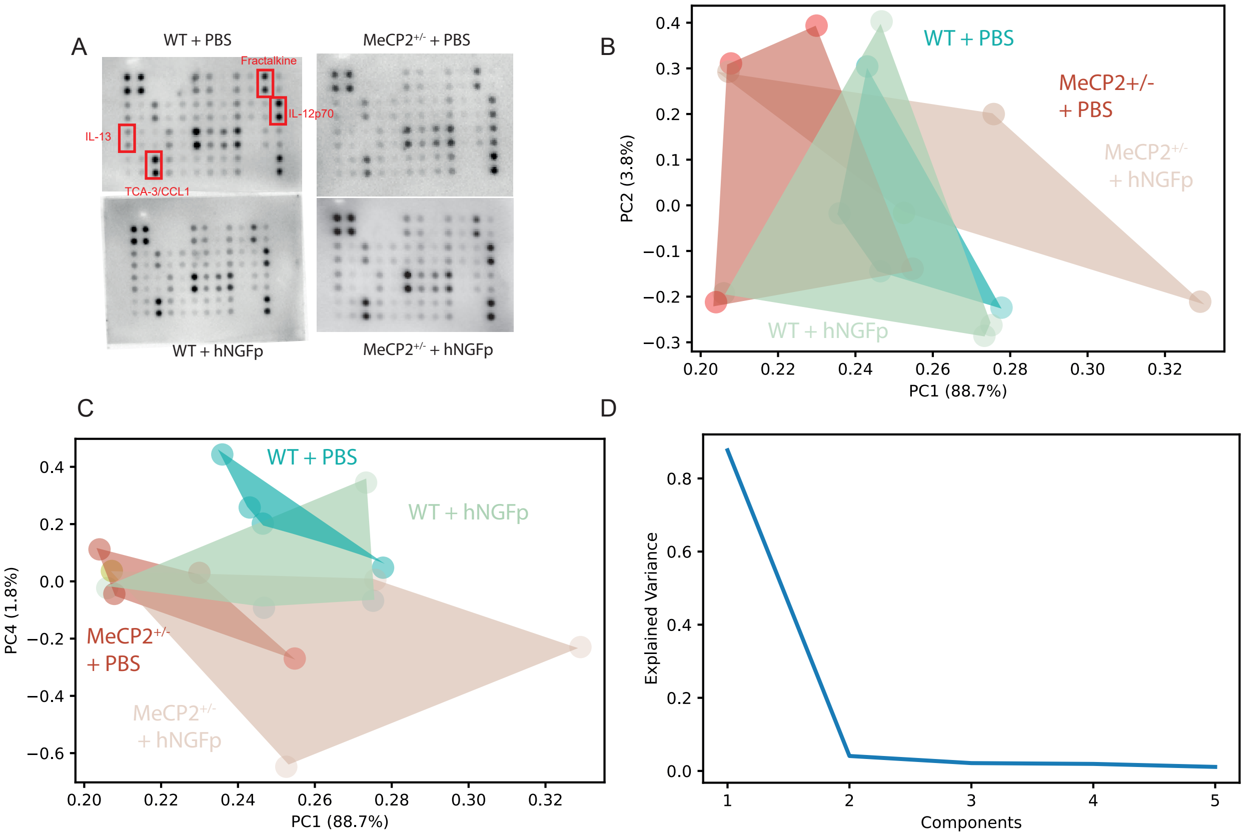

Supplementary Figure 3. Supplementary PCA data for cytokine analysis. (A) Cytokine array membranes for each group analyzed. For the whole list of cytokines see the manufacturer website - https://www.raybiotech.com/c-series-mouse-inflammation-array-1-4/ (B, C) Plot of the PC1/2 and PC1/4 components respectively (n=4 per group). (D) Explained variance for each of the components identified using the PCA package from sk.learn in Python.

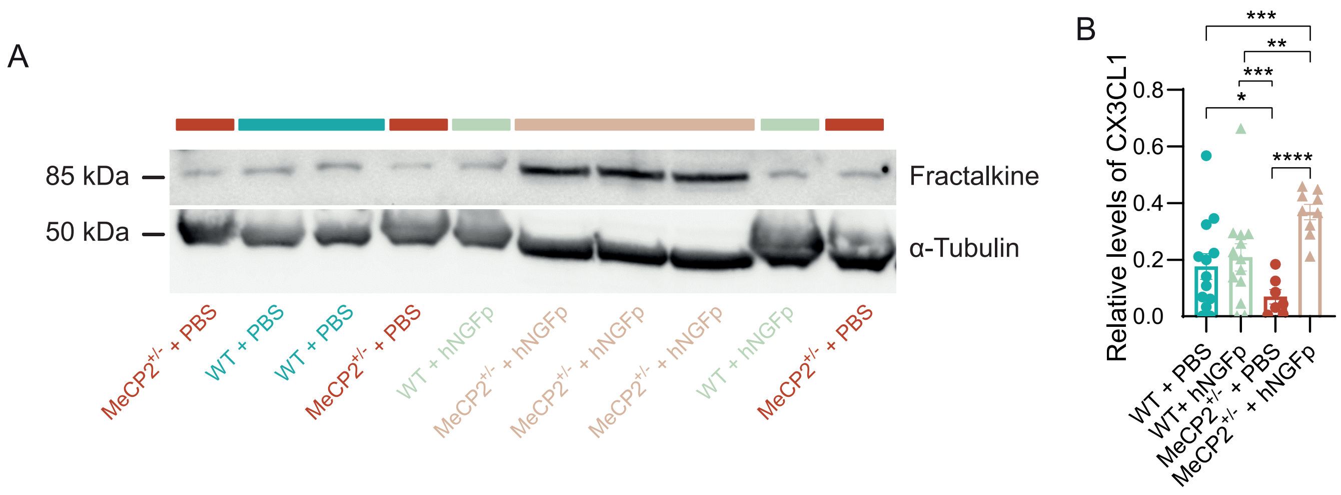

Supplementary Figure 4. Western Blot of fractalkine (A) Blot and (B) Bar plot (Mixed-effects model (REML) & Tukey's multiple comparisons test; Interaction: F (1, 2) = 110.5, p=0.0902; MeCP2+/- + PBS vs. MeCP2+/- + hNGFp p<0.0001; MeCP2+/- + PBS vs. WT + hNGFp p=0.0006; WT + PBS vs. MeCP2+/- + PBS p=0,0116; WT + PBS vs MeCP2+/- + hNGFp p=0.0001; WT + hNGFp + MeCP2+/- + hNGFp p=0.0042; (n=7-13 per group). Mean ± SEM and individual values are reported in all scatter plots with bars.
